# Formulation and Antibacterial Activity Evaluation of Quaternized Aminochitosan Membrane for Wound Dressing Applications

**DOI:** 10.3390/polym13152428

**Published:** 2021-07-23

**Authors:** Ahmed M. Omer, Tamer M. Tamer, Randa E. Khalifa, Abdelazeem S. Eltaweil, Mona M. Agwa, Sally Sabra, Mahmoud S. Abd-Elmonem, Mohamed S. Mohy-Eldin, Zyta M. Ziora

**Affiliations:** 1 Polymer Materials Research Department, Advanced Technology and New Materials Research Institute (ATNMRI), City of Scientific Research and Technological Applications (SRTA-City), New Borg El-Arab City, Alexandria P.O. Box 21934, Egypt; ttamer85@gmail.com (T.M.T.); randaghonim@yahoo.com (R.E.K.); mmohyeldin@srtacity.sci.eg (M.S.M.-E.); 2 Chemistry Department, Faculty of Science, Alexandria University, Alexandria 21526, Egypt; abdelazeemeltaweil@alexu.edu.eg; 3 Department of Chemistry of Natural and Microbial Products, Pharmaceutical and Drug Industries Research Division, National Research Centre, Dokki, Giza 12622, Egypt; mona.m.agwa@alexu.edu.eg; 4 Department of Biotechnology, Institute of Graduate studies and Research, Alexandria University, Alexandria 21526, Egypt; ssabra@alexu.edu.eg; 5 National Organizations for Drug Control and Research (NODCAR), Cairo P.O. Box 29, Egypt; mahmmoudsayed1973@gmail.com; 6 Institute for Molecular Bioscience, The University of Queensland, Brisbane 4072, Australia; z.ziora@uq.edu.au

**Keywords:** quaternization, chitosan derivative, inhibition, biodegradability, antibacterial dressers

## Abstract

Much attention has been paid to chitosan biopolymer for advanced wound dressing owing to its exceptional biological characteristics comprising biodegradability, biocompatibility and respectable antibacterial activity. This study intended to develop a new antibacterial membrane based on quaternized aminochitosan (QAMCS) derivative. Herein, aminochitosan (AMCS) derivative was quaternized by N-(2-Chloroethyl) dimethylamine hydrochloride with different ratios. The pre-fabricated membranes were characterized by several analysis tools. The results indicate that maximum surface potential of +42.2 mV was attained by QAMCS3 membrane compared with +33.6 mV for native AMCS membrane. Moreover, membranes displayed higher surface roughness (1.27 ± 0.24 μm) and higher water uptake value (237 ± 8%) for QAMCS3 compared with 0.81 ± 0.08 μm and 165 ± 6% for neat AMCS membranes. Furthermore, the antibacterial activities were evaluated against *Escherichia coli*, *Pseudomonas aeruginosa*, *Staphylococcus aureus* and *Bacillus cereus*. Superior antibacterial activities with maximum inhibition values of 80–98% were accomplished by QAMCS3 membranes compared with 57–72% for AMCS membrane. Minimum inhibition concentration (MIC) results denote that the antibacterial activities were significantly boosted with increasing of polymeric sample concentration from 25 to 250 µg/mL. Additionally, all membranes unveiled better biocompatibility and respectable biodegradability, suggesting their possible application for advanced wound dressing.

## 1. Introduction

Over the last thirty years, immense interest of researchers has been directed to natural biopolymers and the possibility of expanding their application in various medical and pharmaceutical fields [1,2]. This is due to their outstanding features, including nature availability, tunable biodegradability, low-cost production, biocompatibility and non-toxicity [3,4,5]. Among these, chitosan (CS) is the most plentiful natural polymer in nature after cellulose and comprises randomly distributed (1→4)-linked 2-amino-2-deoxy-β -d-glucopyranose units [6]. Chitosan is the deacetylated form of chitin biopolymer, which is the main constituent of the exoskeleton of crustacean shells such as shrimp, lobsters and crabs [7]. CS possesses mucoadhesive property, excellent blood compatibility and acceptable biodegradability [8]. In addition, the bioactivities of CS also include antimicrobial [9], antioxidant [10], anti-inflammatory [11], anticoagulant [12], anticancer [13], antiviral [14] and antifungal [15] activities. In the last two decades, CS has emerged as a highly imperative marine biopolymer that can be utilized in diverse fields, such as water treatment, enzyme immobilization and fuel cells [16,17,18]. Therefore, CS has been effectually employed in a vast array of extensively diverse biomedical applications, including drug delivery [19], tissue engineering [20] and wound dressing [21]. Under acidic conditions, the present amino groups along the CS backbone undergo protonation (NH_3_^+^), which is responsible for its solubilization and is associated with its antimicrobial activity. It has been reported that CS has the ability to induce fibroblast growth, stop bleeding and stimulate the migration of mononuclear and polymorphonuclear cells, and consequently, boost re-epithelization as well as skin regeneration [22]. In addition, it has the capability to inhibit the attack of several types of microorganisms via the formation of a strong protective film [23]. It has been reported that CS has the ability to accelerate wound healing not only through its basic antimicrobial characteristics but also by the benefit of its aptitude to deliver various antimicrobial agents and growth factors to various types of wounds [24]. Additionally, aminated chitosan (aminochitosan; AmCs) is an interesting chitosan derivative that has been developed by the authors’ previous work with extra amine groups [25]. Compared with neat CS biopolymer, AMCS derivative has demonstrated better biological properties, including biocompatibility and biodegradability [26]. However, the poor solubility of CS in most solvents at neutral and high pH significantly limits its usage. In addition, the antimicrobial activity of CS towards some microorganisms has been repressed due to the non-stop mutations of microorganisms to resist the activities of antibiotics [27]. To address this shortcoming, several physicochemical modifications including grafting [28], crosslinking [29] and Schiff base formation [30], in addition to combination with bioactive molecules [31] and quaternization [32], have accompanied the native biopolymer to improve its activity against a wide-range of microorganisms. Lately, much attention was paid to the quaternization of CS to provide water-soluble CS derivatives via the introduction of a quaternary ammonium moiety on the dissociative -OH^−^ or primary -NH_2_ groups [33].

The quaternization process can meaningfully overcome the poor solubility of CS at the neutral/high pHs, while conserving its positive charge and thus can widen its possible biomedical applications over the entire pH range accordingly [34,35]. Therefore, quaternized CS derivatives possess better antimicrobial activities toward different microorganisms compared with native CS, since the base site of these cationic derivatives is the cytoplasmic film of bacterial cells [36]. It has been reported that different degrees of quaternization display different activities against bacteria [37]. Several quaternized chitosan derivatives, including *N,N,N*-trimethyl chitosan (TMC) [38], hydroxypropyltrimethylammonium chloride CS (HACC) [39], glycidyl trimethyl ammonium chloride(GTMAC)/CS [40] and *N,N,N*-trimethyl O-(2-hydroxy-3-trimethylammonium propyl) chitosans (TMHTMAPC) [41], have been developed, and their antimicrobial activities have been examined. The results revealed that these quaternized derivatives were shown to be fairly effectual as antimicrobial agents against Gram-positive and Gram-negative bacteria such as *Escherichia coli*, *Staphylococcus epidermidis* and *Staphylococcus aureus*. The versatility and adaptability of quaternized chitosan derivatives offer a unique opportunity for the development of new antibacterial agents in addition to the preclusion of infectious diseases.

The current study deals with the continuous development of new antibacterial agents-based chitosan biopolymer. Herein, we aimed to develop new quaternized aminochitosan (QAMCS) membranes as efficient antibacterial membranes for wound dressing applications. Th introduced quaternary ammonium salt as well as the existence of extra amine groups on the AMCS backbone were expected to improve its biological properties, specifically boosting antibacterial activity. The chemical structures, thermal properties and surface morphologies of the fabricated QAMCS membranes were investigated various characterization tools, respectively. In addition, the surface charges, roughness and mechanical properties were explored. The bio-characteristics of the developed membranes, including their antibacterial activities against four kinds of bacteria that usually provoking wound infections as well as their biodegradability and blood-compatibility, were also examined.

## 2. Materials and Methods

### 2.1. Materials

Aminochitosan (AMCS; low viscosity; DD = 92%) was provided by ATNMRI, SRTA-City (Egypt). N-(2-Chloroethyl) dimethylamine hydrochloride (assay 99%), N-methyl-2-pyrrolidinone (assay 98%) and sodium iodide (assay ≥ 99%) were acquired from Sigma-Aldrich Co. (Taufkirchen, Munich, Germany). Acetic acid (assay 98%), ethanol (assay 99%), glycerol (assay 99%) and sodium hydroxide (purity 98%) were brought by El-Nasr Co. (Cairo, Egypt).

### 2.2. Bacterial Strains

Two Gram-negative bacteria (*Escherichia coli* and *Pseudomonas aeruginosa*) and two Gram-positive bacteria (*Staphylococcus aureus* and *Bacillus cereus*) were used for the antibacterial evaluation. Herein, we have selected these types of bacteria as they are considered the most common causative organisms associated with wound infections. The tested bacteria were refreshed before use through inoculating them overnight at 37 °C under constant shaking rate (150 rpm) in Luria–Bertani (LB) culture medium (pH 7), which is composed of peptone (1%), yeast extract (0.5%) and sodium chloride (1%).

### 2.3. Preparation of Quaternized Aminochitosan (QAMCS) Derivative

**Figure 1:**
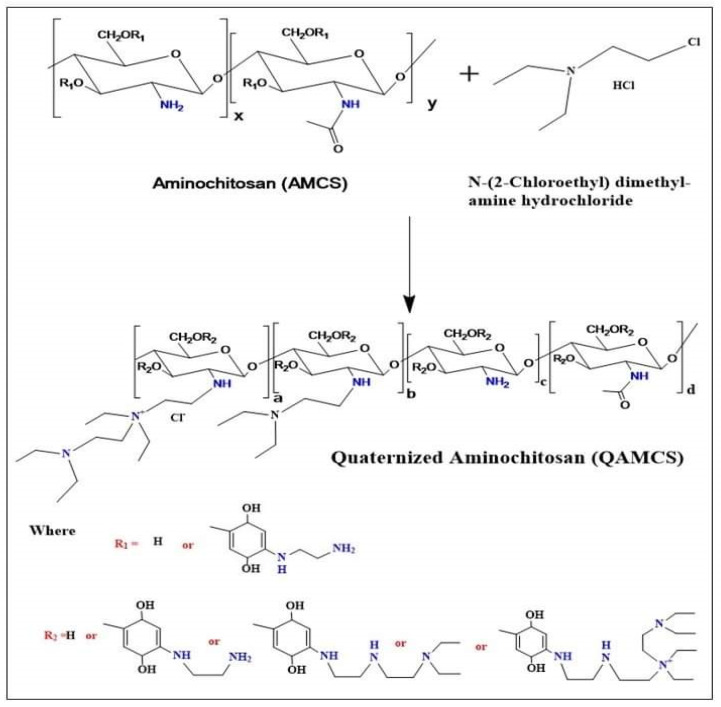
A schematic diagram for the synthesis of quaternized aminochitosan derivative.

The quaternization process was conducted according to the authors’ reported study [26], with a minor modification. Briefly, AMCS (1 g) and NaI (4.8 g; as a catalyst) were placed in a 2-necked flask containing 80 mL of an N-methyl-2-pyrrolidinone/acetic acid (1%) mixture. The reaction was conducted at 60 °C in a shaking water bath till complete solubilization. The flask was connected to a condensation column system. Then, N-(2-Chloroethyl) dimethylamine hydrochloride (as a quaternizing agent) was added with final AMCS: quaternizing agent molar ratios of 1:0.267 (code: QAMCS1), 1:0.534 (code: QAMCS2) and 1:1.06 (code: QAMCS3) under continuous stirring. At the same time, 11 mL of NaOH (15 %; *w*/*v*) was dropwise added while the reaction was contained for 3 h. The obtained product was precipitated by ethanol (200 mL), centrifuged, washed using acetone and finally dried under reduced pressure. Figure 1 displays the mechanism of the preparation of AMCS and QAMCS derivatives.

### 2.4. Membranes Formulation

To formulate membranes, AMCS and QAMCS derivatives were dissolved separately under stirring at 25 °C in acetic acid (2%; *w*/*v*) and distilled water (pH 7.0), respectively, to have final concentration of 2% (*w*/*v*). An accurate 20 mL of the prepared derivatives (including 0.5 mL of glycerol as a plasticizer) was poured into a clean Petri dish (diameter = 7 cm) and left for 48 h at 25 °C to evaporate the solvent. Thereafter, the dried membranes were soaked for approximately 30 s in an aqueous solution of NaOH (1 mol·L^−1^) for neutralization, followed by washing with deionized water. Finally, the wet membranes were rinsed out and fixed to glass plates supported with clamps and allowed to dry at 25 °C until they reached constant weights.

### 2.5. Characterization

The chemical structures of the developed membranes were investigated by Fourier transform infrared spectroscopy (FTIR, Model 8400 S, Shimadzu, Kyoto, Japan). An accurate quantity of tested sample (10 mg) was thoroughly mixed with KBr (spectral purity) at 25 °C, while the absorbance of samples was scanned in the wavenumber range of 500–4000 cm^–1^. In addition, the thermal properties were examined by the Thermogravimetric analyzer (TGA, Model 50/50H, Shimadzu, Japan). TGA analysis was achieved under nitrogen atmosphere (flow rate 40 mL/min), while the temperature was raised gradually from 10 to 800 °C with a constant heating rate (20 °C/min). The morphological characteristics were investigated by a scanning electron microscope (SEM; Model JSM 6360 LA, Joel, Japan) under a voltage potential of 20 kV. Prior to SEM examination, the examined samples were placed on aluminum stubs and coated with a thin layer of gold via a sputter coating system. Furthermore, the surface charges were estimated by Zeta-Sizer (Malvern Panalytical Co., Royston, UK). Surface roughness of all membranes was measured by a surface roughness tester (Model SJ-201P, Kawasaki, Japan). To examine surface roughness, the membrane samples (4 cm × 5 cm) were fixed onto a glass slide with double-sided tap, and the obtained data are presented as the mean average of three measurements.

Additionally, a universal testing machine (AG-1S, Shimadzu, Japan) was employed to explore the mechanical properties of the tested membranes. The tested sample was placed between the grips of the testing machine at constant grip length (5 cm) and speed rate of testing (12.5 mm/min). Finally, the water uptake profiles of membranes were evaluated by soaking of 0.1 g of the tested sample for 24 h in distilled water at 25 °C. Subsequently, the swollen samples were gently separated from the swelling medium and placed between two filter papers to eliminate the excess of water adhering to the surface, which was followed by weighing. Water uptake (*WU*; %) was estimated according to the following Equation (1) [42]:

(1) $$WU\;\left(\%\right)=\frac{W_{f}-W_{i}}{W_{i}}\times 100$$

where *W_f_* and *W_i_* represent the final and initial weights of the tested samples, respectively.

### 2.6. Bioevaluation Studies

#### 2.6.1. Antibacterial Activity Measurements

Several bioassay techniques were applied for screening and evaluation of the antibacterial activity, including well-diffusion, broth or agar dilution and disc-diffusion [43]. The antibacterial activities of the formulated membranes were performed according to the authors’ previous studies [29]. The previously refreshed suspensions of bacteria, i.e., *E. coli, P. aeruginosa, S. aureus* and *B. cereus* (as mentioned in Section 2.2) were diluted for 100 times using the LB broth medium (1%). A portion of 0.1 mL of diluted suspension was cultured in a liquid peptone medium. An accurate quantity of the examined membrane sample (100 µL) was soaked in the diluted suspension and was followed by sterilization at 121 °C for 0.5 h. Next, the inoculated medium was kept under shaking for 24 h at 37 °C. Bacterial growth inhibition (%) was assessed via measuring the absorbance of the culture medium at 620 nm using visible-spectroscopy. The antibacterial activity experiment was implemented in triplicate. The inhibition (%) was calculated according to the following Equation (2):

(2) $$Inhibition\;\left(\%\right)=\frac{A_{a}-A_{b}}{A_{a}}\times 100$$

where *A_b_* and *A_a_* represent the absorbance in the absence and in the presence of the membrane sample, respectively.

#### 2.6.2. Minimum Inhibitory Concentration (MIC) Assay

MIC was conducted to inspect the influence of different concentrations of the developed AMCS and its quaternized form on the growth of the studied bacteria. MIC experiment was performed according to the reported studies using the microtiter plate method [44]. In brief, bacterial strains (*E. coli*, *P. aeruginosa*, *S. aureus* and *B. cereus)* were overnight cultured by incubating them in LB broth under a shaking rate of 150 rpm at 37 °C. Next, bacterial cultures were diluted 100 times using the same LB medium to gain optical densities of 0.9 via measuring the bacterial turbidity at 600 nm. Various concentrations of tested samples (25, 50, 100, 200 and 250 µg/mL) were added into sterile 96-well microplates containing 20 µL of the bacterial culture suspensions. The wells were completed to 200 µL with LB broth free medium and followed by mixing for 2 min at 100 rpm using a bench-shaker. Finally, the wells were left overnight for aerobic incubation at 37 °C. Additionally, the negative and positive controls were prepared separately by mixing the diluted bacterial cultures and the examined samples with the free LB medium, respectively. The microtiter plates were shaken for 30 s using a microplate reader, and the turbidity of bacterial cultures was assayed at 600 nm. The test was conducted in triplicate and the inhibition (%) of microbial growth was calculated according to the following Equation (3):

(3) $$Growth\;Inhibition\;\left(\%\right)=\frac{OD_{a}-OD_{b}}{OD_{a}}\times 100$$

where *OD_a_* and *OD_b_* represent the optical density of normal and inhibited microbial growth, respectively.

#### 2.6.3. Bactericidal Performance Study

To investigate the bactericidal behavior of the developed materials, a microtiter plate approach was employed according to the reported method [45]. Briefly, the overnight cultures of bacterial suspensions were diluted with LB medium to attain optical density equal to 1.2 at 600 nm. An accurate 150 μg of tested sample was added into 1.0 mL of the bacterial suspension. The mixtures were incubated for diverse times (0, 1, 2, 3, 4, 5 and 6 h) at 37 °C. Thereafter, 10 μL of each mixture was injected in 96-well microplates, and the wells were completed up to 200 μL using LB broth. The microplates were then gently shaken and incubated overnight at 37 °C. The turbidities of the bacterial cultures were assessed at 600 nm using a microplate reader. All measurements were performed in triplicate and the results are presented as means and standard deviations (±SD).

### 2.7. Hemocompatibility Studies

Compatibility of the formulated AMCS and its quaternized derivative with human blood was examined according to the formerly reported procedure, with a slight modification [10]. Informed consent was obtained from a volunteer (35 y) before using his blood. In brief, an identified weight of membrane sample (1 cm^2^) was washed by a freshly prepared phosphate buffer solution (pH 7.4) to remove any attached impurities. The tested sample was then placed in a glass test tube comprising an ACD-blood mixture (prepared by mixing 9 mL of fresh human blood with 1 mL of acid citrate dextrose as an anticoagulant), and followed by incubation for 3 h at 37 °C. Concurrently, a control was performed using a free-sample mixture, while deionized water and phosphate buffer were used as positive and negative controls, respectively. After incubation, the tubes were centrifuged at 2000 rpm for 15 min. The optical densities of supernatants (*OD*) at 540 nm were assayed by a UV-spectrophotometer, and the hemolysis (%) was estimated based on the following Equation (4):

(4) $$Hemolysis\;\left(\%\right)=\frac{OD_{s}-OD_{n}}{OD_{p}-OD_{n}}\times 100$$

where *OD*_s_ is the optical density of supernatant in the presence of a studied sample. *OD_n_* and *OD_p_* represent the optical densities of the negative and positive controls, respectively.

### 2.8. Biodegradability Studies

A biodegradability test was performed according to the reported method [29,46]. An exact quantity of the tested sample (0.1 g) was placed in a glass tube containing 10 mL of phosphate buffer (pH 7.0) and lysozyme solution (13 mg L^−1^). The tube was incubated for 24 h at 37 °C. Next, dinitrosalicylic acid (DNS; 1.5 mL) reagent was added carefully to stop the activity of lysozyme enzyme. The mixture was boiled for 15 min and subsequently left to cool. The generated color obtained from the reaction between the DNS reagent with the liberated reduced sugars was analyzed via estimation of the optical density (*OD*) at 570 nm using a visible-spectroscopy.

### 2.9. Statistical Analysis

All experiments were accomplished in triplicates (*n* = 3), and data are presented as means and standard deviations (±SD).

## 3. Results and Discussion

### 3.1. FTIR Analysis

**Figure 2:**
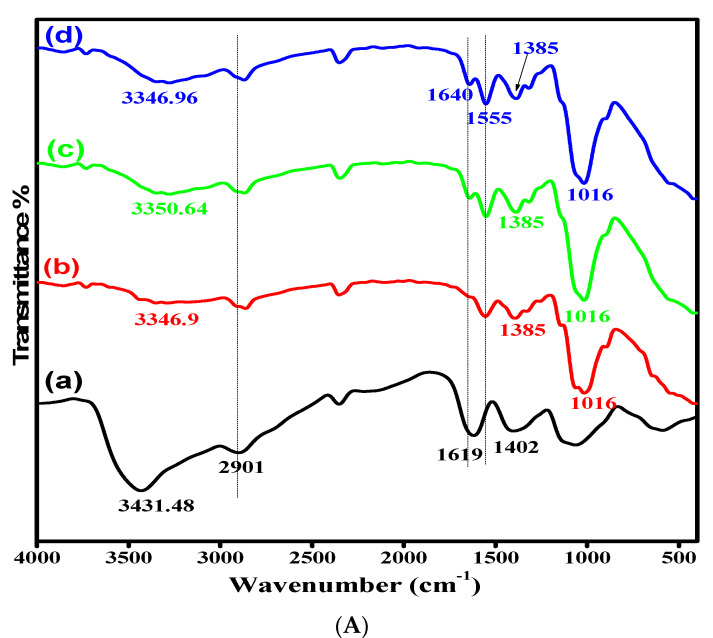

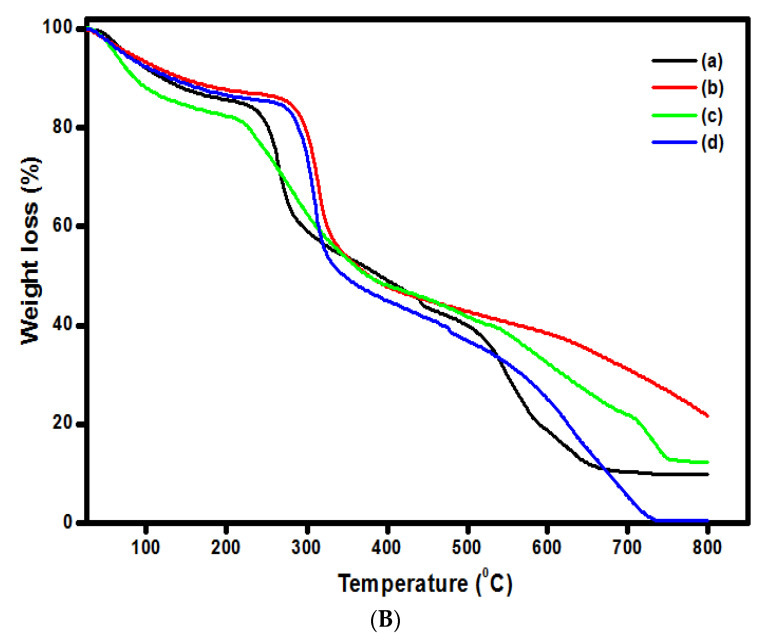
(**A**) FTIR spectra and (**B**) TGA thermograms of (a) aminochitosan (b) quaternized aminochitosan 1 (c) quaternized aminochitosan 2 and (d) quaternized aminochitosan 3 membranes.

The IR spectra of AMCS derivative and its quaternized forms were attained for more details regarding their chemical structures, as shown in Figure 2A. The results obtained clarify that the basic characteristics of the chitosan structure were observed. The spectrum of AMCS showed an absorption band at 3431.48 cm^−1^, which corresponds to the stretching vibration of amine and hydroxyl groups [47]. This band was shifted to the lower frequencies by increasing the quaternization ratio and appeared at 3350.64, 3346.9 and 3346.96 cm^−1^ for QAMCS1, QAMCS2 and QAMCS3, respectively. In addition, the absorption band located at 2901 cm^−1^, which was assigned to the C-H stretching (for methyl and methylene groups), was moved after the quaternization process to 2863–2870 cm^−1^. Moreover, the absorption broad bands at 1619–1640 cm^−1^ and 1555 cm^−1^ corresponded to the stretching vibration of C=O and N–H of amide-I and amide-II, respectively. It was also noted that the N–H bending (1619 cm^−1^) of the primary amine in the AMCS spectrum was significantly affected after the quaternization process since it moved to a higher wavelength (1640 cm^−1^). These observations agree with those reported by other researchers [48,49]. The complex bands at around 1402–1386 cm^−1^ are associated with amide (III), which involves C–N stretching and N-H of the amide linkage. The appearance of multi-peaks at 890–1062 cm^−1^ correspond with C-C, C-O and C-O-C glycosidic bonds. On the other hand, the observed absorption bands around 1016 cm^−1^ in the QAMCS spectra could be attributed to the stretching vibration of C–N in the quaternary ammonium groups. In addition, the peaks at around 1385–1393 cm^−1^ are associated with the C-H symmetric bending of the methyl groups in the generated quaternary ammonium groups [26,50]. Moreover, the peak intensities were amplified with the increase of the quaternization ratio in the membrane matrix, as evidence of the successful preparation process. 

### 3.2. Thermal Properties

The thermal stabilities of the developed membranes were studied in the temperature range from 25 to 800 °C, as shown in Figure 2A and Table 1. The results clarify that the thermal profiles took place in three consecutive stages. The first stage occurred with rising temperature up to 120 °C, with maximum weight losses in the range of 8.38–12.11%. The initial weight loss could be attributed to the elevation of the moisture content from all examined samples [51]. The second weight loss was noticed with increasing temperature up to 350 °C due to the deformation of the C-O-C glycoside bonds as well as decomposition of the pyranose ring [52]. The third decomposition stage was caused by dissociation of adducts, and it was detected with rising temperature beyond 400 °C. The detected degradation stages were matched with the previously reported thermal decomposition profile of chitosan polysaccharide and its derivatives [53]. In addition, at the higher temperatures, the AMCS sample was found to be more stable compared with the QAMCS derivatives, while the thermal stability decreased significantly with increases in the quaternization ratio. It was observed that the temperature required for AMCS sample to lose its half weight (T_50%_ °C) was 391.05 °C compared with 381.62, 376.69 and 347.54 °C for QAMCS1, QAMCS2 and QAMCS3, respectively. These observations could be attributed to the introduction of quaternary ammonium salts into the AMCS backbone, which evidently affects its thermal stability. In all cases, the results confirm that the developed membranes displayed fair thermal stability in the vicinity of human body temperature, which enhances the possibility of their use in advanced wound dressing.

### 3.3. SEM Analysis

**Figure 3:**
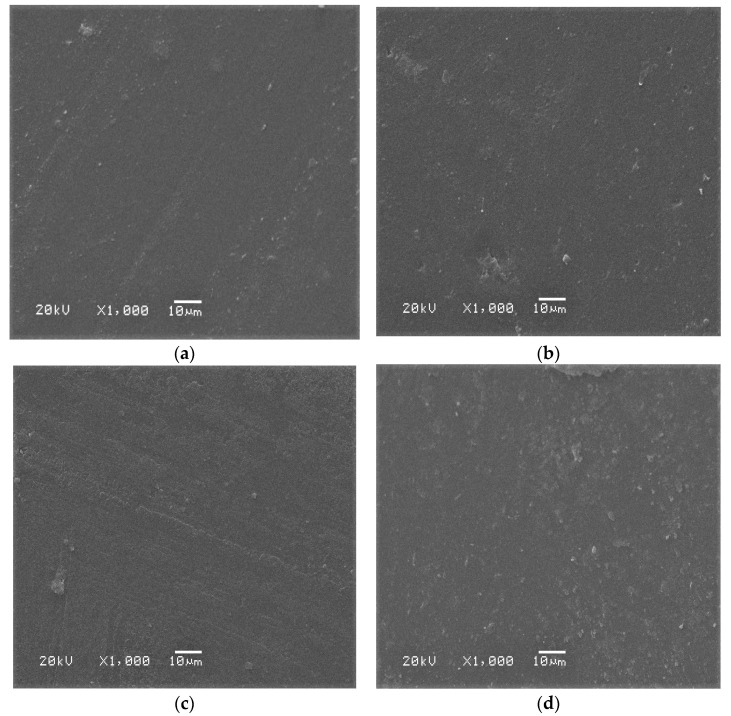
SEM images of (**a**) aminochitosan, (**b**) quaternized aminochitosan 1, (**c**) quaternized aminochitosan 2 and (**d**) quaternized aminochitosan 3 membranes.

Figure 3a–d shows the SEM images of the formulated AMCS and QAMCS membranes. It was observed that AMCS (Figure 3a) displayed a uniform and flat surface except for a few parts that were relatively rough. Meanwhile, the surface morphology of the AMCS membrane was affected significantly after the quaternization process since it changed to a rough surface containing irregular particles and some wrinkles (Figure 3b–d) [29]. The introduction of quaternary ammonium groups distorted the internal order of AMCS chains and disturbed its crystal structure. In addition, the roughness increased with the increasing of the quaternization ratio due to the difference in polarity of both AMCS and the quaternized agent (i.e., N-(2-Chloroethyl) dimethylamine hydrochloride).

### 3.4. Zeta Potentials and Degree of Quaternization

**Figure 4:**
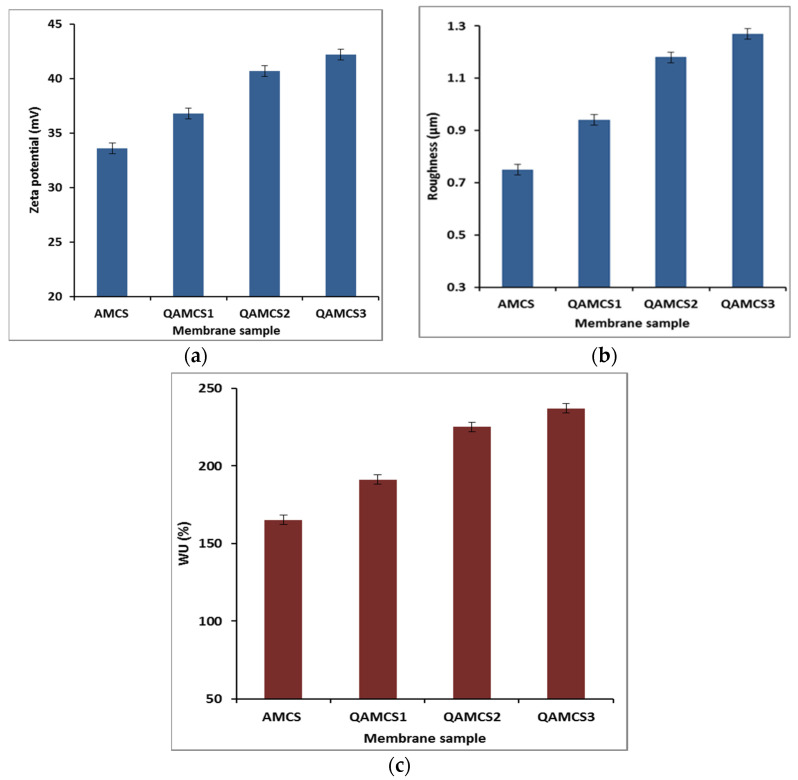
(**a**) Zeta potential, (**b**) roughness and (**c**) water uptake (WU%) values of aminochitosan, quaternized aminochitosan 1, quaternized aminochitosan 2 and quaternized aminochitosan 3 membranes. All measurements were accomplished in triplicate (*n* = 3), and data obtained are expressed as means and standard deviations (±SD).

Figure 4a clarifies that that all the developed membranes possessed positive surface charges. Therefore, with the increase in the quaternization ratio, the zeta potential values increased, as the highest value of +42.2 mV was recorded by QAMCS3 membrane compared with +33.6 mV for the neat AMCS membrane. Additionally, the results also clarify that the degree of quaternization was increased with an increase in the quaternization ratio in the feed mixture. Maximum values of 74.2, 83.3 and 94.7% were recorded and obtained by QAMCS1, QAMCS2 and QAMCS3, respectively. These results could be explained by the formation of more positively charged quaternary ammonium salts with the raising of the quaternization ratio in the membrane matrix. It has been reported that increasing the surface positive charges on the membrane surface strongly enhances the electrostatic attraction forces with the negatively charged surface of the bacterial cell wall. Accordingly, the antibacterial activity of the membranes increases [54].

### 3.5. Surface Roughness

The surface roughness of the formulated membranes was studied as depicted in Figure 4b. The results obtained clarify that the developed QAMCS membranes displayed rougher surface compared with the native AMCS membrane (0.81 ± 0.08 μm). In addition, the membrane roughness was increased gradually from 0.94 ± 0.12 to 1.27 ± 0.24 μm by increasing the quaternized agent ratio from 0.267 M (QAMCS1) to 1.06 M (QAMCS3). These results agree with those observed by SEM analysis (Section 2.3). Additionally, the authors’ previous studies revealed that increasing the membrane roughness might improve the adhesion of the membranes to the cells and tissue. Consequently, the cells’ attachment would be improved since it would be supportive for their use as antibacterial wound dressers and as scaffolds for tissue regeneration to induce adhesion of the dermal fibroblasts [21].

### 3.6. Water Uptake Evaluation

Figure 4c demonstrates the water uptake profiles of the developed AMCS membrane and its quaternized derivatives. The results signify that all developed membranes displayed acceptable water uptake profiles as a result of the existence of hydrophilic -OH and -NH_2_ groups along the AMCS backbone, which can bind with water molecules. Indeed, the water uptake characteristic is considered to be one of the most important features of membrane-based wound dressings. Therefore, wound dressings with high water uptake can effectively provide a moist-wound environment and facilitate the passage of fibroblasts, keratinocytes and endothelial cells to the damaged wound area. Additionally, it can absorb the surplus wound exudates that prompt the wounds to bacterial infections, improve the hemostasis properties and hasten the healing process accordingly [55]. In addition, the results also clarify that the quaternization process greatly boosted the water uptake profiles compared with native AMCS membrane. The highest water uptake (WU) value was achieved by the QAMCS3 membrane as it reached a maximum value of 237 ± 8%, compared with 165 ± 6% for the non-quaternized AMCS membrane. These results could be explained by improvement of the hydrophilic nature of membranes after the generation of more hydrophilic quaternary ammonium groups.

### 3.7. Mechanical Properties

Table 2 presents the mechanical characteristics data for the developed membranes. The results indicate that all tested membranes displayed good mechanical characteristics. An increase in the tensile strength values was observed by increasing the quaternization ratio in a membrane’s matrix. Therefore, a maximum force value of 141 ± 2.6 N and a maximum stress value of 54.6 ± 1.7 N/m^2^ were recorded by the QAMCS3 membrane compared with 125.5 ± 2.1 N and 46.5 ± 1.4 N/m^2^ for the neat AMCS membrane. The decent mechanical properties of these observations were associated with the strong internal attraction forces of membrane compositions, which harvest a rise in membrane rigidity [56]. In addition, the formation of multiple bonds by the ionic functional groups including hydroxyl, amine and the quaternary ammonium groups could also improve the mechanical characteristics of the formulated membranes.

### 3.8. Membrane Bioevaluation

#### 3.8.1. In Vitro Hemocompatibility

**Figure 5:**
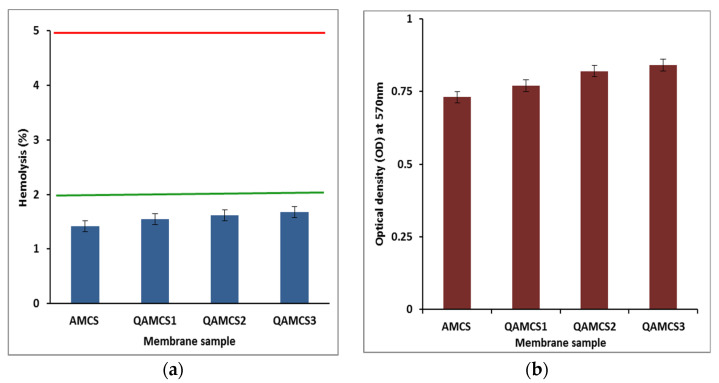
(**a**) Hemocompatibility data and (**b**) biodegradation profiles of aminochitosan, quaternized aminochitosan 1, quaternized aminochitosan 2 and quaternized aminochitosan 3 membranes. All measurements were accomplished in triplicate (*n* = 3), and data obtained are expressed as means and standard deviations (±SD).

The developed membranes were tested their compatibilities as a function of the blood hemolysis (%), and data obtained are depicted in Figure 5a The results signify that all membranes possessed very few hemolysis (%) values below 2%. Blood compatibility is considered one of the crucial assessments needed to examine the potential of wound dressing membranes before their in vivo application. Biomaterials have been classified according to American Society for Testing and Materials (ASTM; F 756-00, 2000; US Pharmacopeia XXIII, 1994) into non-hemolytic (hemolytic index <2%), slightly hemolytic (hemolytic index of (2–5%) and hemolytic materials (hemolytic index over 5%) [57]. Although the increase in the quaternization ratio in a membrane’s matrix has a slight effect on its hemolytic index, all values were found to be in the safe level, which ranged from 1.42 ± 0.75% to 1.68 ± 0.36%. These findings confirm that the developed membranes are biocompatible, i.e., non-hemolytic materials, owing to the admirable biocompatibility nature of the parent chitosan polysaccharide. Similar observations have been reported for wound dressing-based chitosan biopolymers [29,58,59].

#### 3.8.2. In Vitro Biodegradation

Figure 5b displays the biodegradability profiles of AMCS and QAMCS derivative membranes. As expected, all examined membranes were biodegraded in the presence of the lysozyme enzyme. Therefore, the enzyme could be potentially adsorbed by the reactive functional groups in the membrane matrix, and the glycosidic bonds would be hydrolyzed, causing in a degradation of the polymer constituents [19,29]. Furthermore, the biodegradation profile was slightly improved by increasing the quaternization ratio. This is due to the increase in the number of free hydrophilic adsorbing groups, which reflects positively on the biodegradation process.

#### 3.8.3. Antibacterial Activity Evaluation

**Figure 6:**
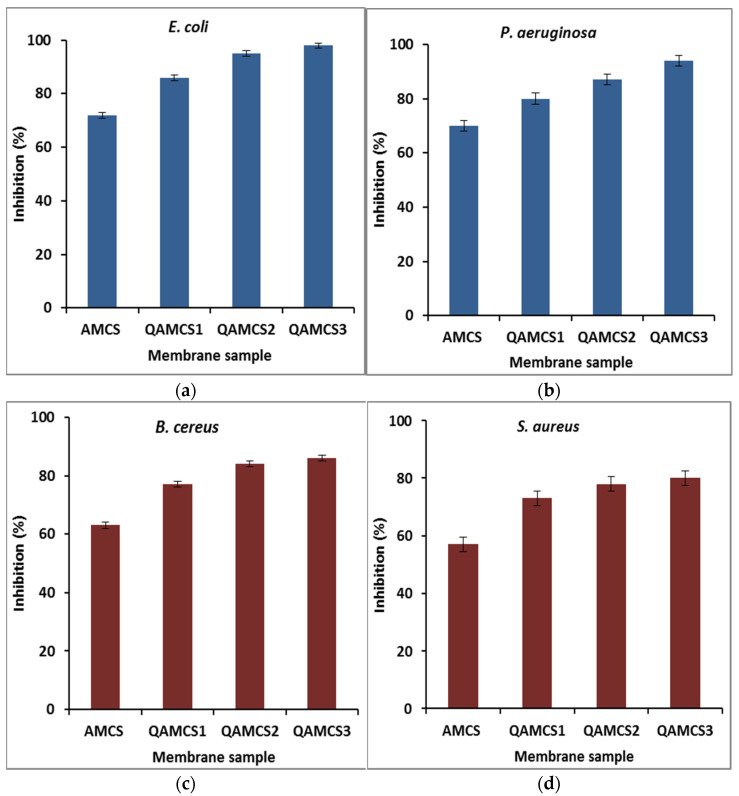
Antibacterial activities of aminochitosan, quaternized aminochitosan 1, quaternized aminochitosan 2 and quaternized aminochitosan 3 membranes against (**a**) *E. coli*, (**b**) *P. aeruginosa*, (**c**) *B. cereus* and (**d**) *S. aureus*. All measurements were accomplished in triplicate (*n* = 3), and data obtained are expressed as means and standard deviations (±SD).

Figure 6a–d demonstrates the antimicrobial activity profiles of the formulated membranes against different four types of bacteria. The results signify that the quaternized AMCS derivatives displayed greater antibacterial activity against all tested bacterium types as compared with pure AMCS sample. Therefore, the highest inhibition (%) values of 98, 95, 86 and 72% were recorded by QAMCS3, QAMCS2, QAMCS1 and the neat AMCS membranes, respectively, and were obtained against *E. coli*. As expected, a significant enlargement in the inhibition (%) was noticed with the rising of the quaternized agent content from 0.267 M (QAMCS1) to 1.06 M (QAMCS3) in the feed mixture. Further introduction of permanent positively charged quaternary ammonium groups on the AMCS backbone dramatically enriched its antibacterial power from 73% to 86% (QAMCS1) to maximum values of 80% to 98% (QAMCS3). Moreover, the highest inhibition (%) values of 98, 94, 86 and 80% were recorded by QAMCS3 membrane against *E. coli*, *P. aeruginosa*, *B. cereus* and *S. aureus*, respectively, while native AMCS membrane recorded maximum inhibition values in the range of 57–72%. Indeed, bacterial infection causes a lot of complications, including the delays in the wound healing process, and it sometimes leads to death. The antimicrobial activities of chitosan/and its derivatives have received substantial attention.

Factually, AMCS has a unique self-antibacterial characteristic owing to its positively charged NH_2_ groups. Although the mechanism of antibacterial action of chitosan/or its derivative is not yet definitely understood, several mechanisms have been proposed to explore this aspect [60]. The most appropriate mechanism to explore the antibacterial activity of AMCS and its quaternized derivatives is mainly associated with the present reactive positively charged NH_2_ (in case of AMCS) and the quaternary ammonium groups (in case of QAMCS). These functional groups have an affinity to interact with the negatively charged outer membranes, specifically for Gram-negative bacteria. Consequently, a leakage of the proteinaceous and other intracellular ingredients (i.e., amino acids, proteins, glucose and lactic dehydrogenase) would occur as a result of the membrane disruption of the bacterial cell [30,61]. In addition, these interactions can extensively alter cell permeability and block of the feeding channels that are responsible for the exchange of electrolytes and nutrients [62]. Accordingly, an inhibition of the normal bacteria metabolism befalls and is followed by the death of the bacterial cells.

Similar observations have been reported with other quaternized chitosan derivatives, which are consistent with the antimicrobial properties obtained in this study. It has been stated that *N,N,N*-diethylmethyl CS exhibits greater antibacterial action against *E. coli* compared with non-quaternized CS [63]. Furthermore, hydroxypropyltrimethylammonium chloride CS (HACC) [39], *N,N,N*-trimethyl O-(2-hydroxy-3-trimethylammonium propyl) chitosan (TMHTMAPC) [64] and *N*-benzyl chitosans (GTMAC) [32] derivatives have been developed with different degrees of quaternization. These quaternized derivatives with high quaternization ratios exhibited higher activity against *Staphylococcus aureus* and *Staphylococcus epidermidis* compared with that obtained by native CS.

#### 3.8.4. MIC Evaluation

**Figure 7:**
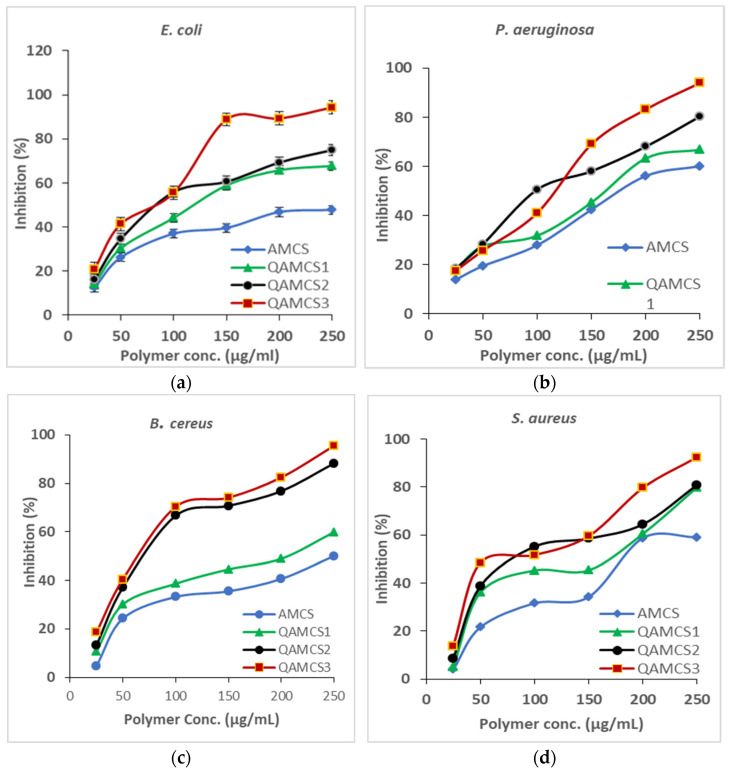
MIC results of different concentrations of aminochitosan, quaternized aminochitosan 1, quaternized aminochitosan 2 and quaternized aminochitosan 3 membranes against (**a**) *E. coli*, (**b**) *P. aeruginosa*, (**c**) *B. cereus* and (**d**) *S. aureus*. All measurements were accomplished in triplicate (*n* = 3), and data obtained are expressed as means and standard deviations. (±SD).

Figure 7a–d shows the MIC results of QAMCS derivative in addition to native AMCS. The results clarify that a dramatic increase in the inhibition percentage (%) was noticed for all studied bacteria (*E. coli*, *P. aeruginosa*, *B. cereus* and *S. aureus*) with raising the quantity of the examined sample from 25 to 250 µg/mL. The minimal concentration (25 µg/mL) of all tested polymeric samples demonstrated various inhibition responses; hence, AMCS recorded 12.44% against *E. coli*, 13.95% against *P. aeruginosa*, 4.65% against *B. cereus* and 3.99% against *S. aureus*. Indeed, MIC could be considered as a practical indicator of primary activity against pathogenic microorganisms.

This assay is essential to examine the antibacterial potency of the new QAMCS derivative compared with neat AMCS, as well as to investigate the vulnerabilities of bacteria to our developed materials. The results also refer to the finding that the inhibition percentage (%) values against both positive and negative Gram bacteria were increased with increasing the quaternization degree as a result of increasing the surface positive charges of the developed membranes. Therefore, the most effective derivative was the QAMCS3 sample, which recorded the maximal values of 20.87% against *E. coli*, 17.5% against *P. aeruginosa*, 18.54% against *B. cereus* and 13.82% against *S. aureus* at the same polymer concentration (25 µg/mL). These findings agree with other studies, which reported that inhibition of bacteria increases with the increase in the surface positive charges [65,66].

#### 3.8.5. Bactericidal Behavior

**Figure 8:**
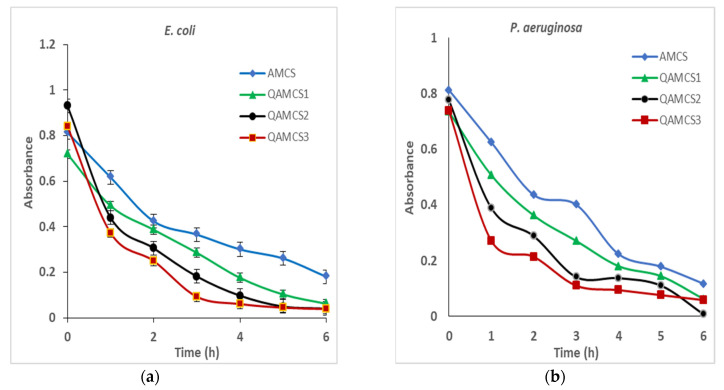

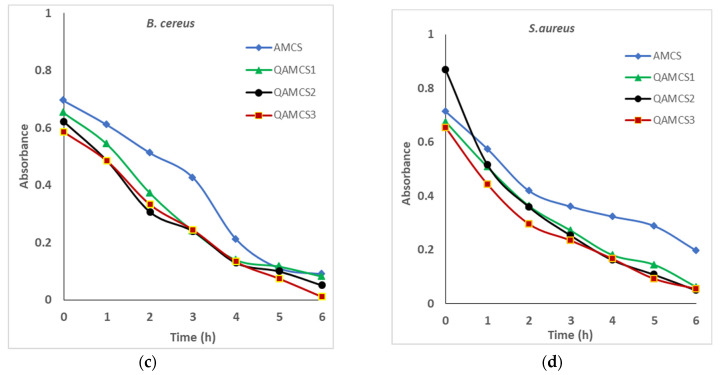
Bactericidal profiles of aminochitosan, quaternized aminochitosan 1, quaternized aminochitosan 2 and quaternized aminochitosan 3 membranes against (**a**) *E. coli*, (**b**) *P. aeruginosa*, (**c**) *B. cereus* and (**d**) *S. aureus*. All measurements were accomplished in triplicate (*n* = 3), and data obtained are expressed as means and standard deviations (±SD).

The developed QAMCS derivatives were assessed for their activities toward the tested pathogenic bacteria, which were previously designated for different periods. Figure 8 clarifies the relationships between the inhibition rate of bacteria (%) and the contact time in the range of 0–6 h. This investigation was necessary to distinguish the modes of bacteria against tested biomaterials in order to control the drug dosage and duration time. Therefore, various factors could affect this examination, such as concentration of the examined materials, conditions of bacterial growth, bacterial density and time.

The obtained results of the bactericidal assay indicated that all tested AMCS and QAMCS derivatives offered bactericidal behavior against bacteria based on the kind of examined polymer and bacteria type. Therefore, the AmCS sample displayed the lowest activity, since the inhibition percentage (%) was going down at the first 2 h in the case of *E. coli*, *P. aeruginosa* and *S. aureus*, and then the activity was slowly reduced. On the other hand, in the case of *B. cereus*, a slower activity was observed at the first 3 h, while the activity rate was faster later. The existence of protein channels within the outer membrane of Gram-negative bacteria might hamper the entrance of AMCS and QAMCS residues into the cells. The presence of extra amine groups in AMCS and its quaternized forms possess more positive charges and consequently damaged bacterial cells. Thus, quaternization of amine groups of AMCS by N-(2-Chloroethyl) dimethylamine hydrochloride generate permanent positive charges on the polymer chains and consequently promote the activity of new QAMCS derivatives against tested bacteria. Similar observations have been reported and have proved that the bactericidal activity of chitosan against *Escherichia coli* (*E*. *coli-*ATCC 25925) was multiplied several times after being converted to *N*-propyl-*N*, *N*-dimethyl chitosan derivative [37,67].

### 3.9. Techno-Economic Aspects and Future Research Directions

Indeed, many millions of people who experience injuries suffer from non-healing wounds. Efficient wound healing needs carefully considered treatment, often necessitating various clinic and hospital visits, and therefore, the ensuing costs of wound dressing materials are confounding. Incessant shadowing is essential to guide a proper dressing for wound infection, and rational usage of antimicrobial materials is required to overcome the complications caused by bacterial wound infections. Since a large amount of the crustacean exoskeleton is readily offered as a by-product of the seafood processing industry, the raw material for chitosan production is fairly inexpensive, and thereby the manufacture of chitosan on a large scale from this renewable bio-resource is economically feasible. Another important aspect to be considered is that utilizing the shellfish waste for chitin production provides a solution to the waste disposal problem and provides an alternative for the use of this oceanic resource. The FDA has approved chitosan for medical uses such as bandages for wound dressing and drug delivery systems. Moreover, one Norwegian company that fabricates shrimp-derived chitosan proclaimed in 2001 that its purified chitosan product achieved self-affirmed Generally Recognized as Safe (GRAS) status in the US market. The growing product application in water treatment, pharmaceutical and biomedical and cosmetics industries is expected to drive market growth. There are several challenges facing researchers regarding wound dressing-based chitosan biopolymer. Among these, prospective studies are crucial in order to augment the antibacterial activities as well as to prove the application feasibility of the developed chitosan derivatives. In addition, more efforts are necessary to develop new low-cost modification techniques for the fabrication of wound dressing-based chitosan and for the evaluation of their efficacies on a large-scale for expected successful field applications. Although quaternized CS derivatives have been considered as effective antimicrobial agents, their modes of action need to be further studied in depth. Additionally, the effect of selective substitutions, O-Substitution and N-Substitution, on the antimicrobial efficiency needs further investigation. Reaching a balance between good antimicrobial activity and low mammalian toxicity from one side and the right DS is cutting edge. Finally, the induction of bacterial resistance by quaternized CS derivatives could limit their use, so the mechanisms of action as well as extensive in vivo studies should also be considered. Finally, more comprehensive economic and market examination studies are required.

## 4. Conclusions

Quaternized aminochitosan (QAMCS) membranes were formulated and characterized by FTIR, TGA and SEM characterization tools. The positive charges on the surface of QAMCS membranes were augmented and reached maximum surface potential of +42.2 mV, with an increase in the quaternized agent ratio of up to 1.06 M, which dramatically enriched its antibacterial power from 73% to 86% (QAMCS1) to maximum values ranging from 80% to 98% (QAMCS3). Moreover, the highest inhibition percentage (%) values of 98, 94, 86 and 80% were recorded by QAMCS3 membrane against *E. coli*, *P. aeruginosa*, *B. cereus* and *S. aureus*, respectively, while native AMCS membrane recorded maximum inhibition values in the range of 57–72%. The quaternization process greatly boosted the water uptake profiles compared with native AMCS membrane. The highest water uptake (WU) value was achieved by the QAMCS3 membrane as it reached a maximum value of 237 ± 8% compared with 165 ± 6% for the non-quaternized AMCS membrane. A maximum force value of 141 ± 2.6 N and a maximum stress value of 54.6 ± 1.7 N/m^2^ were recorded by the QAMCS3 membrane compared with 125.5 ± 2.1 N and 46.5 ± 1.4 N/m^2^ for neat AMCS membrane. In addition, the membrane roughness was increased gradually from 0.94 to 1.27 μm for (QAMCS1) and (QAMCS3) compared with AMCS membrane (0.81 μm,). Testing the blood compatibility of QAMCS membranes showed that their hemolytic index values were detected in the safe level, which ranged from 1.42 ± 0.75% to 1.68 ± 0.36%. These findings confirm that the developed membranes are biocompatible. Moreover, the antibacterial activities were significantly boosted after the quaternization process since the highest inhibition (%) values were obtained by the QAMCS3 sample. MIC and bactericidal assays proved the adequate antibacterial activity of the developed membranes against all selected bacteria. Furthermore, the biodegradation profile was slightly improved with increases in the quaternization ratio. Therefore, QAMCS membranes possess the basic requirements to be potentially applied as antibacterial wound dressing for the acceleration of wound healing.

## Figures and Tables

**Table 1:** TGA data for the developed aminochitosan, quaternized aminochitosan 1, quaternized aminochitosan 2 and quaternized aminochitosan 3 membranes.

Membranes	Weight Loss (%)		T_50%_ °C
	0–120 °C	Up to 350 °C	
AMCS	10.01	46.02	391.05
QAMCS1	8.38	46.26	381.62
QAMCS2	12.11	47.24	376.69
QAMCS3	9.35	50.64	347.54

**Table 2:** Mechanical parameters of aminochitosan, quaternized aminochitosan 1, quaternized aminochitosan 2 and quaternized aminochitosan 3 membranes. Values are expressed as means and standard deviations (±SD; *n* = 3).

Membrane Sample	Max. Force (N)	Max. Stress σ_max_ (N/m^2^)	Max. Strain ʎ_max_ (%)
AMCS	125.5 ± 2.1	46.5 ± 1.4	7.8 ± 1.7
QAMCS1	133.3 ± 2.5	50.1 ± 1.3	12.5 ± 1.2
QAMCS2	136 ± 1.7	51.3 ± 2.3	14.3 ± 1.6
QAMCS3	141 ± 1.4	54.6 ± 1.7	15.9 ± 1.2

## Data Availability

The data presented in this study are available on request from the corresponding author.

## Sample Availability

Samples are available from the authors.
